# Visualization Techniques of Time-Oriented Data for the Comparison of Single Patients With Multiple Patients or Cohorts: Scoping Review

**DOI:** 10.2196/38041

**Published:** 2022-10-24

**Authors:** Jan Scheer, Alisa Volkert, Nicolas Brich, Lina Weinert, Nandhini Santhanam, Michael Krone, Thomas Ganslandt, Martin Boeker, Till Nagel

**Affiliations:** 1 Human Data Interaction Lab Mannheim University of Applied Sciences Mannheim Germany; 2 Center for Innovative Care University Hospital Tübingen Tübingen Germany; 3 Big Data Visual Analytics in Life Sciences Institute for Bioinformatics and Medical Informatics University of Tübingen Tübingen Germany; 4 Institute of Medical Informatics Heidelberg University Hospital Heidelberg Germany; 5 Abteilung für Biomedizinische Informatik Zentrum für Präventivmedizin und Digitale Gesundheit Baden-Württemberg Medizinische Fakultät Mannheim der Universität Heidelberg Mannheim Germany; 6 Chair of Medical Informatics Friedrich-Alexander-Universität Erlangen-Nürnberg Erlangen Germany; 7 University Hospital Rechts der Isar School of Medicine Technical University of Munich München Germany

**Keywords:** patient data, comparison, visualization systems, visual analytics, information visualization, cohorts, multiple patients, single patients, time-oriented data

## Abstract

**Background:**

Visual analysis and data delivery in the form of visualizations are of great importance in health care, as such forms of presentation can reduce errors and improve care and can also help provide new insights into long-term disease progression. Information visualization and visual analytics also address the complexity of long-term, time-oriented patient data by reducing inherent complexity and facilitating a focus on underlying and hidden patterns.

**Objective:**

This review aims to provide an overview of visualization techniques for time-oriented data in health care, supporting the comparison of patients. We systematically collected literature and report on the visualization techniques supporting the comparison of time-based data sets of single patients with those of multiple patients or their cohorts and summarized the use of these techniques.

**Methods:**

This scoping review used the PRISMA-ScR (Preferred Reporting Items for Systematic Reviews and Meta-Analyses extension for Scoping Reviews) checklist. After all collected articles were screened by 16 reviewers according to the criteria, 6 reviewers extracted the set of variables under investigation. The characteristics of these variables were based on existing taxonomies or identified through open coding.

**Results:**

Of the 249 screened articles, we identified 22 (8.8%) that fit all criteria and reviewed them in depth. We collected and synthesized findings from these articles for medical aspects such as medical context, medical objective, and medical data type, as well as for the core investigated aspects of visualization techniques, interaction techniques, and supported tasks. The extracted articles were published between 2003 and 2019 and were mostly situated in clinical research. These systems used a wide range of visualization techniques, most frequently showing changes over time. Timelines and temporal line charts occurred 8 times each, followed by histograms with 7 occurrences and scatterplots with 5 occurrences. We report on the findings quantitatively through visual summarization, as well as qualitatively.

**Conclusions:**

The articles under review in general mitigated complexity through visualization and supported diverse medical objectives. We identified 3 distinct patient entities: single patients, multiple patients, and cohorts. Cohorts were typically visualized in condensed form, either through prior data aggregation or through visual summarization, whereas visualization of individual patients often contained finer details. All the systems provided mechanisms for viewing and comparing patient data. However, explicitly comparing a single patient with multiple patients or a cohort was supported only by a few systems. These systems mainly use basic visualization techniques, with some using novel visualizations tailored to a specific task. Overall, we found the visual comparison of measurements between single and multiple patients or cohorts to be underdeveloped, and we argue for further research in a systematic review, as well as the usefulness of a design space.

## Introduction

### Overview

The digitization of health care processes has led to large volumes of digitized patient data, enabling new scenarios for data analytics and visualization. In addition to other forms of data representation, visual representation is becoming increasingly important for describing and analyzing data, as well as for drawing conclusions from data and making decisions based on them. The forms of visual representation are as diverse as the data, stemming from all areas of the health care system, such as the care of patients in various subareas of inpatient medicine, such as internal medicine or surgery, emergency and intensive care, and outpatient medicine. The presentation of individual patient data is as important as the presentation of the aggregated data of groups of individuals with certain characteristics. Thus, it has become increasingly important to present individual patient cases in such a way that they are comparable with each other or with cohorts. This goal becomes even more tangible as visualization systems enable the visual analysis of complex, high-dimensional, and heterogeneous data for different objectives.

Although visualization systems for electronic medical record analysis have been developed for decades, most health care information systems still lack basic information visualization concepts. However, visual analysis and delivery of data in the form of visualizations are of great importance in health care, as such forms of representation can reduce errors and improve care [1] and can also help provide new insights into long-term disease trajectories. Information visualization and visual analytics also address the complexity of long-term, time-oriented patient data by reducing the inherent complexity and facilitating a focus on underlying and hidden patterns [2]. Visualization techniques for temporal data enable clinicians to quickly identify relevant trends in patient health records. Visual comparison techniques help clinicians look for differences between a particular patient’s data and his or her group, allowing them to identify, for example, whether treatment needs to be adjusted. In a research context, exploratory data visualization for hypothesis generation is a well-established approach to cohort analysis [3]. By using appropriate interactive visualization techniques, both established and emerging, clinicians and researchers can effectively and efficiently detect patterns, explore relationships, and identify anomalies.

Visualization of time-oriented data is a well-researched area of information visualization across diverse domains, such as finance, the environment, and life sciences. A book by Aigner et al [4] reports on 101 different visualization techniques for time-oriented data and is, to the best of our knowledge, the most detailed review in this area. The research and design of information visualization is a user-centric area and has led to frameworks proposing a classification for a what-why-how differentiation [5]. Munzner [6] suggested a general approach for task-, data-, and user-driven visualization design, which is currently a widespread method in the visualization community and can be applied to time-oriented data [7]. On the basis of the given aspects of data and time (what), as well as user objectives and associated tasks (why), different approaches to visualization techniques (how) have been described [4].

With the increasing availability of clinical patient data for secondary use in clinical research, new opportunities for longitudinal studies and data analyses are emerging. Existing studies have captured time-oriented data visualization in health care [4,8]. However, these reviews do not specifically focus on comparing individuals with other individuals or cohorts. However, the largest and most important task associated with all available data is comparison, for example, with earlier periods of a patient’s journey, other similar multiple patients, or a cohort. We anticipate that this task will become increasingly important and diverse in the future.

However, there appears to be a gap in research specifically related to these visual comparison tasks. Rind et al [9] identified this as an open challenge. In their systematic review, they reported on time-oriented data visualization techniques in health care and pointed out the lack of research on the comparison of a single patient to a group of patients with similar histories. For this reason, we specifically address the visualization techniques used to compare single patients with multiple patients or with a cohort and report on the differences and gaps in design for single-patient, multiple-patient, and whole-cohort visualization. Although information visualization and visual analytics are well-established fields and their application in the medical field has been explored for decades, the use of interactive visualizations for the analysis of patients and their cohorts is still a very active area of research. Therefore, we have gathered works from both scientific fields, the medical informatics and visualization community, to provide a comprehensive overview of the state of the art.

This review aims to answer the following research questions (RQs):

- RQ1: Which visualization techniques are used to compare time-oriented patient data with their cohort data?
- RQ2: What visual analysis objectives and tasks are being supported?
- RQ3: What are the characteristics of the visualization systems and applications?

The goal of this study was to provide an overview of visualization techniques for time-oriented data in health care, which support patient comparison. More specifically, we systematically collected literature and report on the interactive visualization techniques that support the comparison of time-oriented data sets of a single patient with those of multiple patients or their cohorts and summarized the use of these techniques. The visualization systems are described according to their medical characteristics, data type categories, and further relevant visualization aspects of such interactions.

### Background

#### Visually Analyzing Data With Information Visualization and Visual Analytics

Historically, the field of visualization research has been divided into 3 subfields: scientific visualization, information visualization, and visual analytics. Although this division is currently sometimes considered too arbitrary and outdated, it helps to structure different techniques and applications. Scientific visualization deals with data that have an inherent spatial reference, such as volume data from medical imaging or atomic coordinates in a molecule. The terms “information visualization” and “visual analytics” are often used synonymously, although they are not synonymous. However, the division is often less clear: *information visualization* represents abstract data in a visual context and expresses patterns or trends that are inherent to the data (using mostly 2D visualization methods, eg, line or bar charts). Information visualizations are often interactive, enabling the manipulation of data or the visualization for in-depth analysis. *Visual analytics* also represents data in an interactive visual context but further supports the discovery and identification process of patterns and trends by combining automated analysis with interactive visualizations; that is, visual analytics systems use information visualization methods to communicate data. Moreover, additional aids for facilitating the understanding of rather complex data are provided and, therefore, are able to support decision-making. The term *visual analytics* was originally coined in 2005 in the context of complex data analysis systems for homeland security [10], where it was already described broadly as “the science of analytical reasoning facilitated by interactive visual interfaces.” Currently, the visual analytics approach is used in many different application areas, ranging from security over software analytics to biology, medicine, and health [11]. Often but not necessarily, it applies machine learning methods to support data analysis.

As it is often unclear whether a system should be classified as interactive information visualization or visual analytics, the reviewed visualization systems covered both areas. Especially in the health sector, time-oriented data visualizations play an important role, both on the level of individual or multiple patients and on the level of entire populations or cohorts; therefore, they are important subjects of research in information visualization and visual analytics. Although existing reviews investigate visual analytic methods and techniques in public health and report on techniques from an epidemiological point of view [8], or focus on visual analytic methods and techniques applied to public health and health services research [12], these review visualizations for populations do not specifically address the visual analysis of single patients.

#### Investigating Patients: Individual Patients, Multiple Patients, and Cohorts

Historically, the first cornerstones in the field of exploring data visualizations of individual patients were laid in *LifeLines* by Plaisant et al [12]. Numerous visualization systems for electronic patient records or their data analysis have been developed since, such as Knowledge-based Navigation of Abstractions for Visualization and Explanation (KNAVE) [13], KNAVE-II [14], Visualization of Time-Oriented Records [15], LifeLines 2 [16], EventFlow [17], and CareCruiser [18] to name a few. Tools and concepts supporting the visual analysis of patient progression and cohort comparisons are still under active investigation. A recent example is a visual analytics approach that uses dimensionality reduction to summarize and compare individual participants. This method was used to transform intensive care unit data from a controlled animal experiment into 2D curves representing the changing status of participants, with the possibility of characterizing the ensembles of the participants [19]. Another recent study [20] investigated the visual analysis of event sequences in the context of several topics, of which health care constitutes only a minor portion. Research on the applicability of the other approaches to the health care domain is not covered and, thus, constitutes an avenue for future research.

Existing systematic reviews report on the prevalence of electronic health record (EHR) visualization techniques for individual patients and multiple patients [9,21,22]. Most such visualization systems support the task of analyzing either a single patient or multiple patients. Depending on the context and goal of the analysis, multiple patients with increasing numbers can build up to a cohort. Time-oriented patient data comprise event sequences of different data types, which may be categorized (eg, numeric outcomes to categories) or aggregated in time. The same holds for multiple patients. However, time-oriented cohort data (as in epidemiology) differ in that abstract characteristics such as life expectancy or self-reported outcome measures, for example, pain scales, are used for analyses. These data are often reported, for example, as a calculated mean or median across a group of individuals at specified time points.

#### Comparing Time-Oriented Patient Data

Comparison is a widely supported task in interactive visualization systems [23]. When visually analyzing patient data, the task of comparison is a common part of the process, ranging from comparing information about a single patient to comparing treatment responses at different times and comparing patients in a cohort.

Beyond the context of clinical research and patient care, an increasing number of patients want to manage their own EHRs, analyze their disease progression, and compare it with similar patients, similar to the web-based platform *patientsLikeMe* [24].

However, comparison is not a single clearly defined task but a range of tasks [23]. Brehmer and Munzner [5] specified 3 tasks (or scopes) for the user goal to query a specific target: “identify, compare, and summarize.” This is to query within the scope of a single target (identify), multiple targets (compare), or a set of targets (summarize).

The visualization of time-oriented data of a single or multiple patients has been widely explored [9], and most techniques used for visualizing the data of a single patient can be applied to multiple patients up to a certain degree. However, the visualization of cohorts is different in terms of how data are aggregated in time and value. For example, cohort data in clinical trials comprise data that are usually provided at specific time points (number of visits or days aligned for a baseline event) and, in most cases, are represented as statistical values (eg, mean or SDs).

Comparisons within single patients or within cohorts may seem trivial as the same visualization technique is applied for each. However, this may not be the case for comparing a single patient with a cohort, as both may be visualized using a different technique.

Thus, visual comparisons can be supported in various ways. However, the visualization of multiple records can produce visual complexity when visualizing an excessive number of patient records. Similar to Munzner [6], the survey by Gleicher et al [23] emphasizes the exploration of designs for the information visualization of complex data objects, such as graphs, tabular data, and surfaces, and proposes a general taxonomy of visual designs for comparison. Both works differentiate between 3 types of visual comparisons, namely, juxtaposition (or separation), superposition (overlay), and explicit encoding (explicit representation of the relationships), as well as a combination of these. Juxtapositioning means displaying 2 elements that are the subject of the comparison next to each other, whereas superposition means showing them on top of each other in the same view. Gleicher et al [23] found that comparison tasks became more difficult with more complex data objects and when more objects are to be compared, whereas abstracting the data *before* the comparison can simplify the task.

## Methods

### Protocol and Registration

This scoping review was conducted in accordance with the PRISMA-ScR (Preferred Reporting Items for Systematic Reviews and Meta-Analyses extension for Scoping Reviews) approach. We drafted the protocol for our review by following the checklist in the study by Tricco et al [25]. As this scoping review reports primarily on visualization techniques rather than on the outcomes of medical studies and PROSPERO (International Prospective Register of Systematic Reviews) does not accept scoping reviews, the protocol has not been registered and published.

### Eligibility Criteria

On the basis of the presented objective and RQs, we developed criteria for articles to be eligible for review. Articles need to report on a visualization technique, visualization system, or design study supporting the visual analysis of time-oriented patient data to compare a single patient with multiple patients or a patient cohort.

The inclusion criteria were as follows: (1) articles on visualization techniques of time-oriented patient data; (2) articles on systems, applications, or prototypes to support the visual analysis of time-oriented patient data; (3) implementation of tasks to support the visual analysis of time-oriented patient data; and (4) study of a visualization technique for time-oriented data in which physicians or clinical researchers have undergone a test (or questionnaire).

The exclusion criteria were as follows: (1) articles not in English; (2) articles not focused on abstract time-oriented patient data, for example, medical imaging methods (eg, positron emission tomography, magnetic resonance tomography, functional magnetic resonance imaging, and computed tomography); (3) articles on 3D visualizations supporting surgery, operations, or other medical interventions, for example, augmented or virtual reality applications; and (4) articles on deep learning and other machine learning approaches (using patient data), where visualization is solely used to present the implementation.

Articles focusing on medical imaging methods were excluded as they did not fit the information visualization aspect. Although these use imaging methods and are sometimes called (scientific) visualization, they do not visualize abstract time-oriented patient data.

The criteria were revised during the screening process, and we specified the comparison aspect more strictly to exclude articles on visualizations where comparing single patients to a cohort was not supported, either explicitly or implicitly.

### Information Sources

To collect potentially relevant articles, we searched the following publication databases: PubMed, IEEE Xplore, ACM Digital Library, and the Web of Science core collection. We identified 4 major areas that reflected the concepts of our RQs: time, visualization, data, and health care. Starting with these, we drafted the main sets of keywords based on terms and synonyms from the literature. Along with an experienced librarian, we further refined our search strategy. The full search queries for the different databases are provided in Multimedia Appendix 1. The search was performed on July 2, 2020. The search results were imported into Citavi reference management software. Duplicates were removed after import.

### Search

The initial search strategy was developed by a librarian from the library of the Medical Faculty Mannheim of the University of Heidelberg and was aimed at searching only titles and abstracts. It contained the 4 aspects mentioned earlier: time, visualization, data, and health care. The search strings were reviewed and improved through 3 iterations, and 4 of the reviewers approved the final search strategy. The final search string for PubMed is shown in Textbox 1.

Details of searches on the other databases can be found in Multimedia Appendix 1.

In addition to the database searches, we identified the following reviews regarding the visualization of time-oriented health care data: West et al [21], Preim and Lawonn [8], and Aigner et al [4].

We examined the reference lists of these reviews and identified 13 articles that we considered a fit for our search but were not included in our search results. To address the search for potential gray literature, we included articles from IEEE VIS annual meetings and workshops on time or sequence visualizations.

**Search aspects and PubMed search string.**

**Time**

- (“temporal data”[tiab] OR “temporal sequence*”[tiab] OR “temporal pattern*”[tiab] OR “temporal abstraction*”[tiab] OR “temporal event*”[tiab] OR “time sequence*”[tiab] OR “time series”[tiab] OR “time period*”[tiab] OR “time frame*”[tiab] OR “timeframe*”[tiab] OR timeline*[tiab] OR time-oriented[tiab] OR (“time”[tiab] AND “events”[tiab])) AND

**Visualization**

- (visuali*[tiab] OR “visual analy*”[tiab]) AND

**Data**

- (data[tiab] OR information[tiab]) AND

**Health care**

- (patient[tiab] OR patients[tiab] OR “health care”[tiab] OR health care[tiab] OR cohort*[tiab] OR “electronic health record*”[tiab])

### Selection of Sources of Evidence

In the first screening step, 16 reviewers working in groups of 2 independently screened titles and abstracts for eligibility. In this first screening, we only focused on visualizations of time-oriented patient data and did not include criteria for the comparison of a single patient with a cohort. Disagreements were resolved through discussion and consensus of a third reviewer.

In accordance with our objectives, we discussed the results of the screening and continued to perform a second screening step to apply the criteria of single-to-multiple or cohort comparisons.

The titles, abstracts, and full texts of the remaining articles were skimmed for eligibility for an in-depth full-text analysis. The remaining publications were used for data extraction.

### Data-Charting and Extraction Process

For the data extraction, a data-charting form was developed and refined throughout several iterations. The initial form included several categories and abstractions for meta-information, medical context, data, and visualization aspects.

The form was tested by 4 reviewers by applying it to 2 randomly selected articles, of which 1 was assigned to each reviewer. We discussed our findings for corrections and reconciliations throughout the iterative process and released the final version of the form.

### Data Items

For each of the included articles, we specifically focused on four major aspects: (1) *meta-information* of the article (authors, year, and digital object identifier), (2) *medical characteristics* (disease, medical context, and medical objective), (3) *data type categories* (type of medical data, data type, temporality, temporal spread, and availability of data set), and (4) *visualization aspects* (visualization technique, tasks, interactions, comparison, and evaluation). We extracted several data items for each aspect.

The meta-information was collected from the respective literature databases. It requires no further categorization but can be used to sort and compare publications, for example, by author or year and venue of publication.

The section on *medical characteristics* comprises Medical Subject Headings (MeSH) terms for the diseases and medical objectives. The medical context was clinical research, clinical care, or both. For the extraction and grouping of medical data types, we used the following categories: encounter (or transfers or movements), diagnosis, procedure, laboratory results, medication, cardiology findings, activity, condition, clinical note, treatment plan, tumor severity, survival, Framingham Risk Score, and patient-reported outcome. The categories were based on hierarchically high-ranked concepts of clinical terminology in the MeSH thesaurus and their frequency in the included studies.

The data *type categories* were further distinguished as qualitative, quantitative, categorical, and free-text. Data temporality was determined for the time primitives (single time points, time intervals, or both) and temporal arrangement as either sequential or cyclic. The temporal spread was extracted as short (from hours up to a few days), long (longer than a few days), and short to long (from hours to several days). Data availability was either described as yes (including restricted availability), no (if not available), or not applicable if no further information was given.

In the context of *visualization*, we applied Visual Vocabulary [26] to the visualization techniques found in the studies. Visual Vocabulary aims to improve chart literacy for people outside the visualization research community. This visual overview classifies visualization techniques by their main objective and structures them into 9 categories such as part to whole or correlation. The category of *change over time* is particularly relevant to our investigation of temporal patient data and contains techniques such as line charts, calendar heatmaps, or *Priestley timelines*. The latter shows sequential and parallel events on a temporal x-axis, is similar if not synonymous to Gantt and span charts, and is often simply labeled as “event timeline.” Although Visual Vocabulary is not a standardized taxonomy, it is used in both academia and practice. Other reviews of visualization techniques [27] used the taxonomy proposed by Borkin et al [28], which is a mix of basic graphs, data, and task-oriented categories but does not include time as a specific category. Wilke [29] discussed temporal data visualizations but did not include them in his Directory of Visualizations.

Visualization systems are intended to support a wide set of tasks ranging from simple ones such as finding the laboratory value of a specific patient at a given date to more complex tasks such as comparing the progression of multiple characteristics of all patients within a cohort. To discuss the similarities and differences of such diverse tasks, different frameworks of abstract task descriptions have been proposed.

The extraction of tasks in our review was based on the widely used taxonomy for task abstractions by Brehmer and Munzner [5], whereas the extraction of actions and targets relied on the taxonomy by Munzner [6].

At the top level of the taxonomy by Munzner [6], visualization systems can be categorized according to the user objectives and associated tasks—why users use visualization techniques in terms of actions and targets. Actions can be classified as *analyze*, *search*, and *query*, and these can be further split into subcategories. Regarding visualization systems of the category of *analyze*, a distinction can be made among, for example, offering data analysis for viewing, understanding information, and creating new information. Consuming information includes the discovery of new insights based on visualized data (*Analyze: Consume: Discover*), as well as using the visualization for presenting insights to others (*Analyze: Consume: Present*) [6].

We opted not to use the health data–specific task taxonomy by Theis et al [30] as it is designed to capture tasks from the perspective of patients. The data-driven taxonomy by Rostamzadeh et al [31] provides a framework for activities and tasks at different levels of granularity (activities, subactivities, tasks, and subtasks) and proposes 3 major categories: interpretation, monitoring, and prediction. However, comparison is not explicitly defined as a task but rather mentioned as an inherent task between interpretation (overview: visually compare) and prediction (recognize: similarity). Consequently, we considered this taxonomy unsuitable for the collection of comparison tasks. Therefore, we applied the taxonomy by Gleicher et al [23] for comparison.

We focused on comparison tasks between different types of relationships: single-to-single patient comparison (1-1), single-to-multiple patients comparison (1-n), single-to-cohort comparison (1-1), and cohort-to-cohort comparison (1-1 or 1-n). “Single to single” comparison means that users can compare individual patient data over time with a nominal or target value or with another single patient. “Multiple patients” stands for ≥2 patients with similar traits or characteristics and, in contrast to cohorts, are an ad hoc group (ie, a dynamically selected subset of patients). This includes comparing data, such as time points and time intervals for procedures, diagnosis, laboratory values, and encounters, across patients. The data are often aggregated, as is the case in flow-based or stage-based approaches (eg, Guo et al [32]). By contrast, cohorts are patient collectives in a clinical or academic research setting; that is, cohorts generally include more patients than “multiple patients.” These data tend to be 1D for ≥1 group. This could be averaged over the entire cohort.

More details about the items and their concrete sets of attributes are available in Multimedia Appendix 2 [15,16,18,33-51].

### Critical Appraisal of Individual Sources of Evidence

We critically appraised the individual sources specifically for the comparison task. Visual comparisons can be made between ≥2 individual patients, between patients and a cohort, and between cohorts. During the charting process, we systematically collected and collectively discussed visualization techniques in studies in which the applicability to the RQs was in doubt. Included articles that mentioned both visualization and comparison but did not suit our RQs, for instance, because of the comparison of 2 visualization systems, were excluded from the collection. For uncertain cases, in which the explicit comparison of single patients against a cohort was not clearly provided, we critically evaluated whether the visualization technique could implicitly or potentially facilitate that objective.

### Synthesis of Results

For the synthesis of results, evidence is presented in the form of charts and tables. For the aforementioned data items, we present and justify our selection of terms, schemas, and taxonomies for different relevant attributes to extract. We combined top-down and bottom-up methods in an iterative approach and adapted and refined the terms where necessary.

We aimed to specifically report on the collected visualization techniques and interactions for the comparison task, as well as summarize the disease, medical objective, and the corresponding medical data types.

For the quantitative analysis, we created charts for articles according to the publication year. We used different tools for the analysis, ranging from a simple dashboard tool for preliminary analysis [52] to Jupyter notebooks, using the data analysis library Pandas and the visualization library Altair for exploratory data analysis. The resulting visualizations were also used to inform the qualitative analysis of the selected characteristics of the articles.

## Results

### Selection of Sources of Evidence

**Figure 1:**
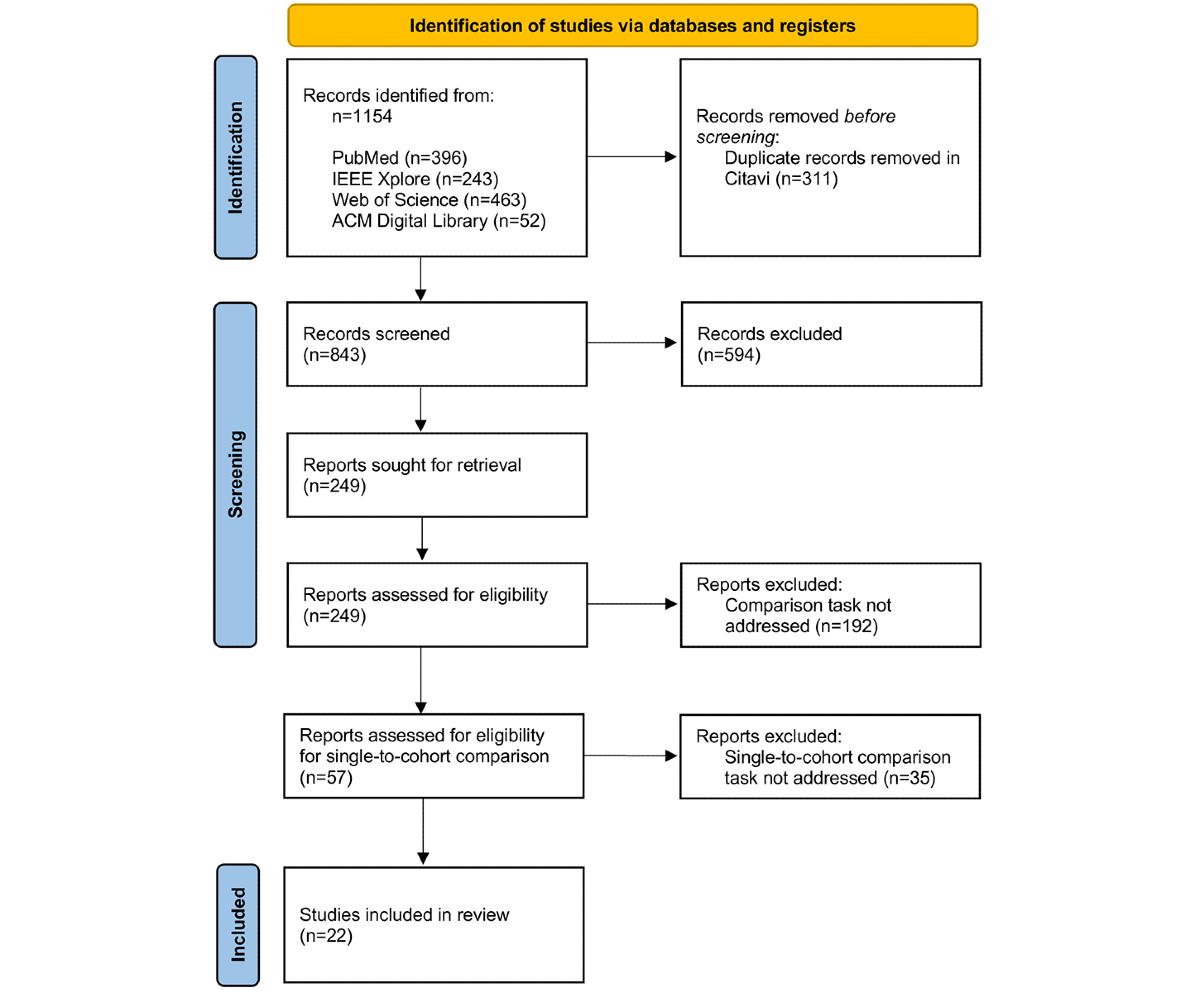
PRISMA (Preferred Reporting Items for Systematic Reviews and Meta-Analyses) flow diagram showing the identification, screening, and inclusion of articles.

We identified 1154 articles through individual database searches. Following separate imports into the reference management software (Citavi) for each database, we removed 26.95% (311/1154) of duplicates electronically. As a first screening step, the titles and abstracts of the 73.05% (843/1154) remaining articles were checked by 16 reviewers, with each article being screened independently by 2 reviewers. Approximately 70.5% (594/843) of papers were excluded based on the study's inclusion and exclusion criteria. In the second screening step, of the 843 articles, we skimmed the full text of 249 (29.5%) articles, of which 192 (22.8%) were excluded as they did not report on the task of comparing patients or cohorts. In the following review step, the full texts of the remaining 57 articles were analyzed in depth for the comparison task, of which 35 (61%) articles were removed as the task of comparing a single patient to multiple patients or a cohort was not provided explicitly, implicitly, or potentially. Of the 843 articles, 22 (2.6%) were included in the synthesis. The PRISMA (Preferred Reporting Items for Systematic Reviews and Meta-Analyses) flow diagram is presented in Figure 1.

### Characteristics of Sources of Evidence

#### Overview

We included 22 articles in the scoping review. Most (17/22, 77%) of the included articles were explicitly either single-to-cohort or single-to-multiple comparisons and implemented a comparison visually. The remaining articles (5/22, 23%) were single-to-single (1/5, 20%) or either cohort-to-cohort or multiple-to-multiple (4/5, 80%) comparisons. We included these 5 articles as the presented techniques, in our opinion, could potentially be applied or extended easily to handle a single-to-cohort or single-to-multiple comparison.

All included articles were published between 2003 and 2020. Of the 22 articles, 8 (36%) were published before the review by Rind et al [9] in 2013.

#### Medical Context

We looked at the medical setting in which the visualization research took place. Medical context in the corpus was mainly clinical research (13/22, 59%), clinical care only (4/22, 18%), and both areas (5/22, 22%; Table 1).

**Table 1:** Medical context in the selected articles (N=22).

Medical context	Studies	Articles, n (%)
Clinical care	Atherton et al [45], Klimov and Shahar [15], Wang et al [16], and Borhani et al [38]	4 (18)
Clinical research	Gschwandtner et al [18], Gotz and Wongsuphasawat et al [41], Stubbs et al [35], Tao et al [46], Gotz et al [42], Cho et al [47], Browne et al [48], Dabek et al [49], Kamaleswaran et al [40], Gomov et al [39], Wildfire et al [34], Nickerson et al [50], and Polack et al [37]	13 (59)
Clinical research or clinical care	Guo et al [43], Rogers et al [36], van Dortmont et al [33], Magallanes et al [44], and Dahlin et al [51]	5 (22)

#### Disease

Most of the included articles (9/22, 41%) reported on pathological conditions, signs, and symptoms. The second most frequent diseases mentioned were related to wounds and injuries (2/22, 9%), neoplasms (2/22, 9%), or cardiovascular disease (2/22, 9%).

#### Medical Objective

Nearly all reviewed articles were found to have “Treatment Outcome” (16/22, 73%) as the primary medical objective. The second most frequent and equally distributed were “Patient Outcome Assessment” (3/22, 14%) and “Disease attributes” (3/22, 14%).

### Results of Individual Sources of Evidence

#### Data

The medical data types identified for visualization and patient comparison contained in the included sources of evidence ranged from the laboratory (13/22, 59%), vital signs (9/22, 41%), and procedures (8/22, 36%) to diagnosis (8/22, 36%). An overview of all extracted data types is provided in Table 2.

The temporal spread of the data was extracted as short (for a couple of hours to less than a few days), long (for more than a few days), and short to long (for data ranging from hours to more than a few days). Most articles reported on either a long (10/22, 45%) or short to long (9/22, 41%) temporal spread, and one of the articles reported on a short (1/22, 4%) spread only. In 4% (1/22) of articles, we could not determine the temporal spread of the data.

**Table 2:** Medical data types in included articles.

Medical data types	Studies	Articles, n (%)
Laboratory	Atherton et al [45], Klimov and Shahar [15], Wang et al [16], Borhani et al [38], Gschwandtner et al [18], Stubbs et al [35], Gotz and Stavropoulos [42], Browne et al [48], Gomov et al [39] Wildfire et al [34], Guo et al [43], van Dortmont et al [33], and Magallanes et al [44]	13 (59)
Vital signs	Borhani et al [38], Stubbs et al [35], Cho et al [47], Browne et al [48], Gomov et al [39], Wildfire et al [34], Nickerson et al [50], Polack et al [37], and van Dortmont et al [33]	9 (41)
Procedures	Wang et al [16], Gotz and Wongsuphasawat [41], Stubbs et al [35], Tao et al [46], Gomov et al [39], Guo et al [43], Rogers et al [36], van Dortmont et al [33], and Dahlin et al [51]	8 (36)
Diagnosis	Stubbs et al [35], Tao et al [46], Gotz and Stavropoulos [42], Dabek et al [49], Gomov et al, 2017 [39], Guo et al [43], van Dortmont et al [33], and Dahlin et al [51]	8 (36)
Medication	Gotz and Wongsuphasawat [41], Gotz and Stavropoulos [42], Browne et al [48], Gomov et al [39], and Guo et al [43]	5 (23)
Encounters (or transfers or movements)	Wang et al [16], Dabek et al [49], Guo et al [43], van Dortmont et al [33], and Magallanes et al [44]	5 (23)
Patient-reported outcomes (or outcomes)	Atherton et al [45], Gotz and Wongsuphasawat [41], Stubbs et al [35], Nickerson et al [50], and Rogers et al [36]	5 (23)
Cardiology	Stubbs et al [35], Kamaleswaran et al [40], Polack et al [37], van Dortmont et al [33], and Gotz and Wongsuphasawat [41]	5 (23)
Activity	Browne et al [48], Nickerson et al [50], Polack et al [37], and Rogers et al [36]	4 (18)
Conditions	Tao et al [46], Dabek et al [49], and Rogers et al [36]	3 (14)
Clinical notes	Gotz and Wongsuphasawat [41] and van Dortmont et al [33]	2 (9)
Other	Gschwandtner et al [18] and (treatment plans) Dahlin et al [51] (tumor severity and survival)	2 (9)

#### Visualization Techniques

**Figure 2:**
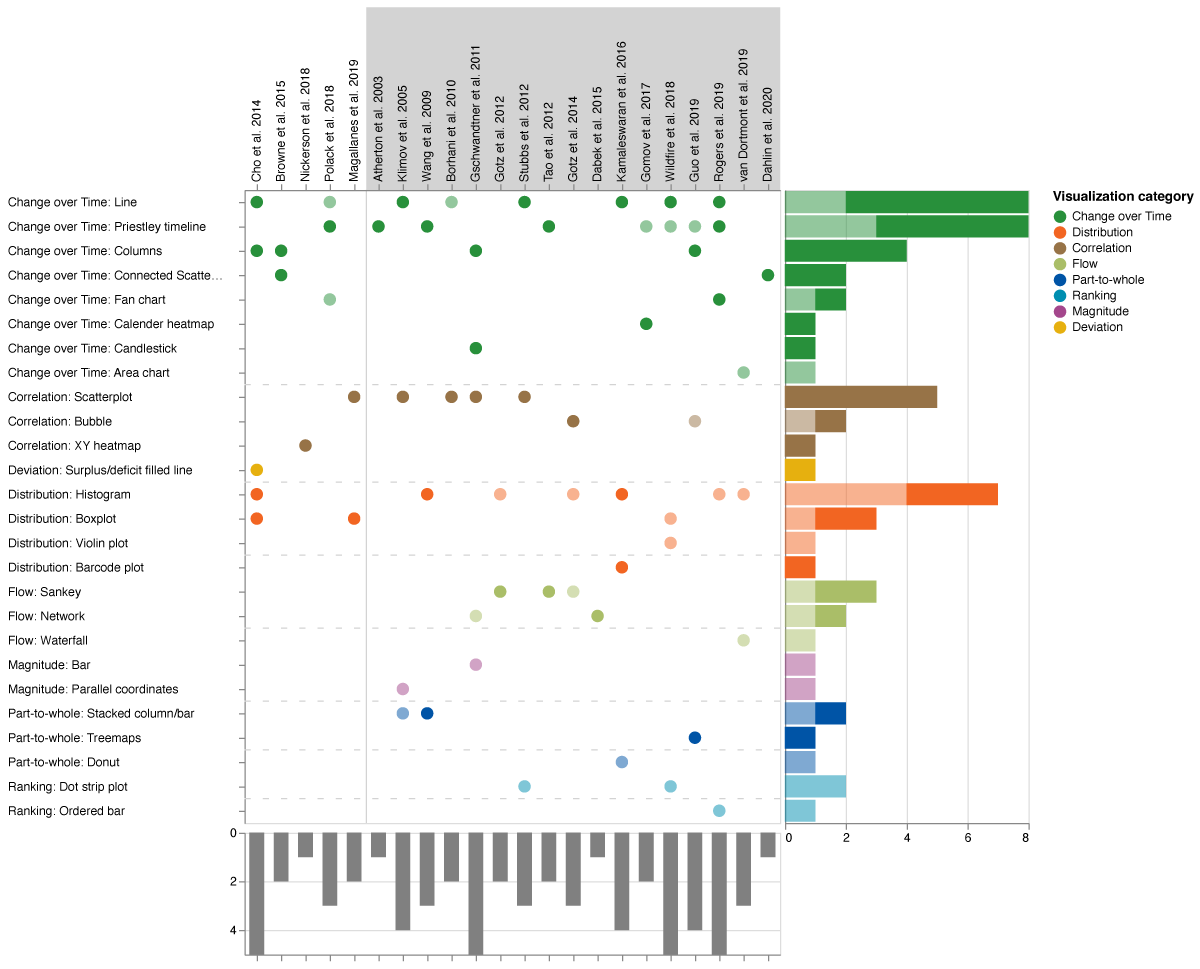
Visualization techniques in the selected articles. Each dot indicates the existence of the technique in a system, with full saturated dots representing the application of the technique for the explicit task of comparison. References within the gray background were identified to support the comparison of multiple patients (single-to-cohort or single-to-multiple). Colors indicate the visualization category, with the bars on the right showing the distribution of the techniques. Bars at the bottom represent the number of techniques identified for each article. Techniques are sorted based on the number of occurrences, and articles are sorted based on the year of publication.

The visualization system comprised ≥1 visualization technique (mean 2.86, SD 1.36). Most (18/22, 82%) of the articles combined multiple visualization techniques, although some of the used techniques were not explicitly directed at the comparison task and were rather used for auxiliary visualizations or for purposes not related to the comparison. Some articles (4/22, 18%) implemented only 1 visualization technique, whereas some (4/22, 18%) featured more complex visualizations using a combination of up to 5 techniques. Systems provide multiple techniques by showing them either side by side (juxtapositioned, eg, in coordinated multiple views or in a dashboard), overlaid (superpositioned, ie, resulting in combined visualizations), or on different pages within a system (eg, interactively switching between multiple views). We identified all visualization techniques and grouped them according to what they mainly intended to show (see the visualization categories in Figure 2).

In general terms and detached from the restriction of analyzing only the articles with explicit single-to-cohort or multiple comparisons, the major visualization techniques identified in our review are line and *Priestley timeline* charts, histograms, scatterplots, and bar charts.

Out the 22 articles, 17 (77%) included at least one technique to show *change over time*, with 2 (9%) articles [36,37] using 3 techniques from this group and 6 (27%) articles using 2 techniques. Overall, we identified 27 occurrences to visualize the progression of 1 or multiple attributes. The most frequently used were temporal line charts (8/22, 36%) and event timelines (8/22, 36%), followed by columns (4/22, 18%), connected scatterplots (2/22, 9%), and fan charts (2/22, 9%). Techniques used once ranged from calendar heat maps to area charts and candlesticks.

The second largest visualization category was *distribution*, with 41% (9/22) of articles having ≥1 technique from this group. Of the 22 articles, overall, we extracted 12 occurrences of techniques showing the distribution of values: histograms were used in 7 (32%) articles, box plots in 3 (14%), and violin plots and barcode plots in 1 (4%) article each.

The third largest category supports the analysis of *correlation*, with scatterplots (5/22, 23%) and bubble charts (2/22, 9%) being the most applied techniques.

Other techniques used more than once included Sankey charts (3/22, 14%) from the *flow* category, bubble charts, network diagrams, stacked bars, and dot strip plots (2/22 each, 9%) from various other categories.

Using the taxonomy for visual comparison by Gleicher et al [23], we found that most works either applied *juxtaposition* (10/22, 45%), some (4/10, 40%) of which featured an additional *explicit encoding* of the relationship, or *superpositioning* (10/22, 45%), some (3/10, 30%) of which featured *explicit encoding*. Only 9% (2/22) of studies applied both *juxtaposition and superposition*, and only a single study applied an additional *explicit encoding*.

#### Tasks

**Figure 3:**
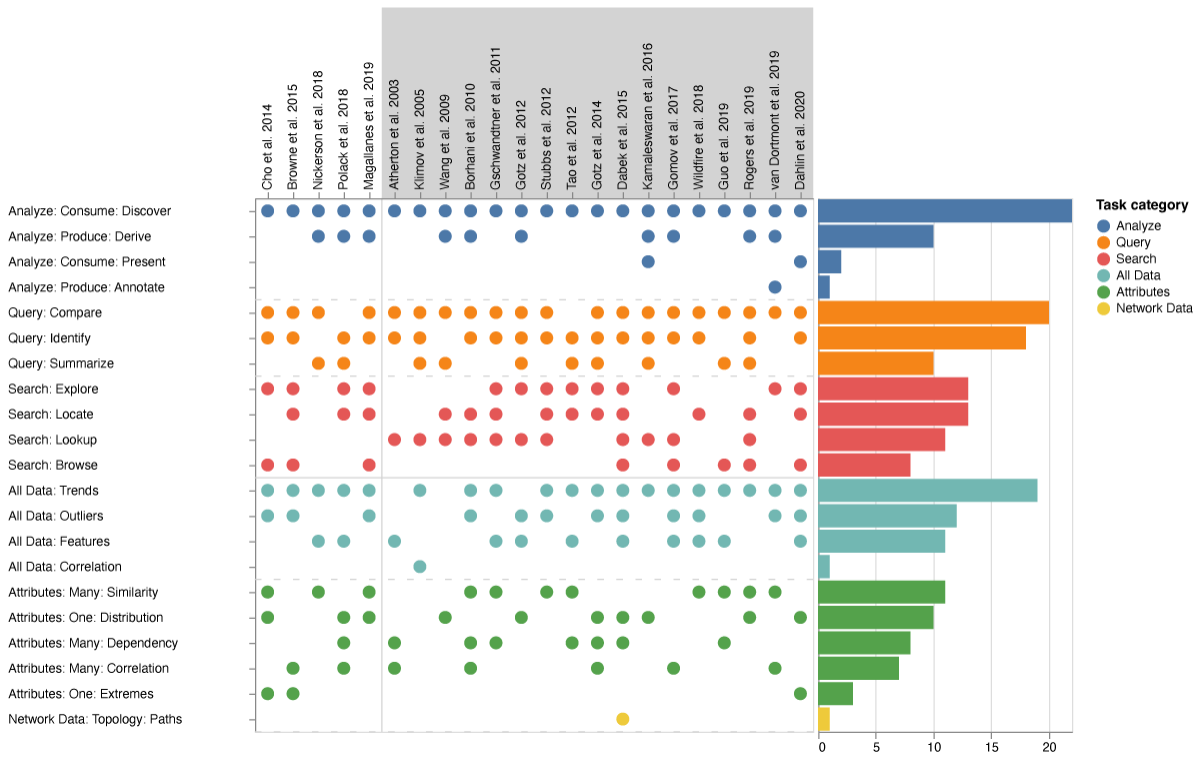
Identified tasks (actions and targets) in the included articles. The plot shows the tasks as actions (analyze, query, and search) and targets (all data, attributes, and network data) that could be completed by visualization systems presented in the articles in our selection. For the categorization of tasks, we used the taxonomy by Munzner [6]. Gray backgrounds indicate articles where patients (single-to-cohort or single-to-multiple) could be compared. Bars on the right-hand side represent the number of articles that used the displayed task category.

User objectives can be characterized by *task* pairs of *actions* and *targets* (compare data items). We have summarized these for the articles in Figure 3.

In all the tasks of the reviewed articles, the action of discovering new knowledge in the visualized data is presented (*Analyze: Consume: Discover*). Only 9% (2/22) of articles featured the action of presenting visualized data as the main action (*Analyze: Consume: Present*).

The second most frequent action in this category was *derive*, creating new material from the shown data (*Analyze: Produce: Derive*; 10/22, 45%). Only one of the articles supported annotation (*Analyze: Produce: Annotate*): the ChronoCorrelator supports tagging events with free-form texts that can be used later on, for example, to highlight or filter events for further exploration [33]. In the category of *search*, the actions *locate* (13/22, 59%), finding a known target at an unknown position*,* and *explore* (13/22, 59%), searching for an unknown target at an unknown position, appeared most frequently. The actions of *lookup* (11/22, 50%), looking for a known element at a known location, and *browse* (8/22, 36%), browsing for ≥1 element without knowing their identity but knowing their characteristics, appeared less frequently. The most frequent action from the category of *query* was *compare* (*Query: Compare*; 20/22, 91%), comparing multiple targets, a result we expected because of our RQ, followed closely by the task of *identify* (*Query: Identify*; 18/22, 82%). The least frequent was the action of *summarize* (*Query: Summarize*; 10/22, 45%).

The targets of the actions were heterogeneous, with a notable exception being *All Data: Trends* (19/22, 86%), which appeared more frequently than others. Targeting outliers (*All Data: Outliers*) and features (*All Data: Features*) was part of 54% (12/22) and 50% (11/22) of articles, respectively. However, correlation as a target (*All Data: Correlation*) was only featured once (1/22, 4%). Actions can target ≥1 attribute of data. The most frequent attribute from the category many was similarity (*Attributes: Many: Similarity*; 11/22, 50%), followed by dependency (*Attributes: Many: Dependency*; 8/22, 36%) and correlation (*Attributes: Many: Correlation*; 7/22, 31%).

The targeting of a single attribute occurred less frequently with distribution (*Attributes: One: Distribution*; 10/22, 45%), appearing far more often than extremes (*Attributes: One: Extremes*; 3/22, 14%).

#### Interaction Techniques

**Figure 4:**
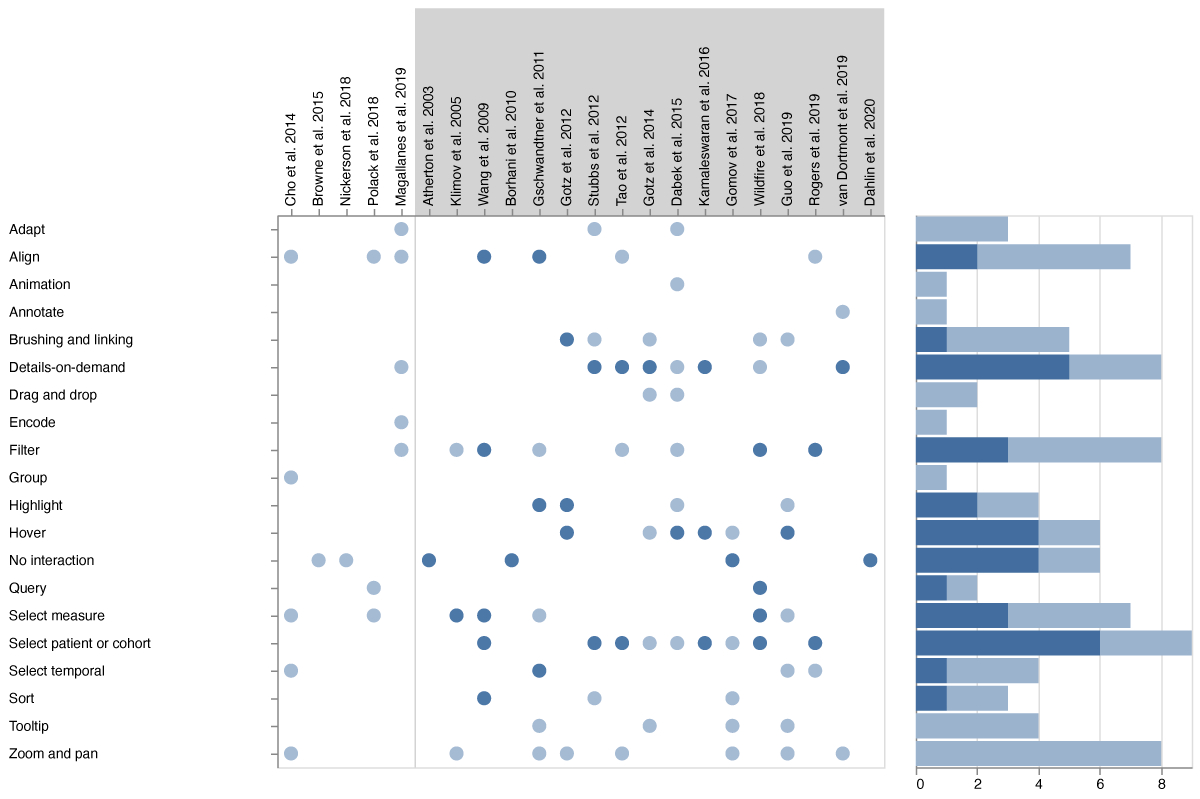
Interaction techniques identified in all included articles. Dark blue dots indicate the explicit application of interaction for the task of comparison. Light blue dots indicate the existence of interactions in the system. References within a gray background were identified to support the comparison of multiple patients (single-to-cohort or single-to-multiple).

Although interactions offer a way of facilitating a more explorative method of data analysis, more than one-quarter of the articles of interest (6/22, 27%) did not offer any interaction (“No interaction” in the middle of Figure 4) regarding the main task—the comparison of single patients with multiple patients or cohorts.

The most frequent interaction possibility found in 41% (9/22) of the articles was the interactive selection of the individual patient or the composition of the cohort. In addition, approximately half of the articles (10/22, 45%) offered an interactive way of showing additional information (details on demand; 8/22, 36%), using hover (6/22, 27%), highlighting (4/22, 18%), or other techniques. The other most commonly used interactions were align (7/22, 32%), filter (8/22, 36%), select measure (7/22, 32%), and zoom and pan (8/22, 36%).

In most studies in scope comparing a single patient with a cohort or multiple patients (17/22, 77%), we identified the selection of a patient and the definition of a cohort as a key interaction technique (*select patient or cohort*; 6/17, 35%).

This was followed by *details on demand* (5/17, 29%) and *hovering* (4/17, 23%). Only 23% (4/17) of these studies did not use any interaction technique for the comparison task.

Depending on the objective of the visualization system to explicitly explore, analyze, and compare patient data, up to 4 interaction techniques are applied directly to support the task for single-to-single or single-to-cohort comparisons [34]. Approximately half of the included articles (9/22, 40%) supported only a single interaction technique for the main task of comparison, whereas some (8/22, 36%) articles combined ≥2 interaction techniques to support the comparison of data elements.

#### Individual Results of Visualization Techniques for Comparisons

##### Overview

In this section, the individual results of some of the included articles are presented. The most frequent visualization techniques are shown, and a more detailed analysis is provided for line charts, Priestley timelines, scatterplots, and histograms.

For the visual comparison of time-oriented patient data from the category of change over time, line charts and Priestley timelines were used most frequently. The second most commonly used visualization categories were distribution and correlation, with histograms and scatterplots. Depending on the complexity of the presented visualization system, between 1 and 5 visualization techniques were used to visually support a comparison (Figure 2). Table 3 summarizes the visualization techniques used for comparison, the supported combinations of patient entities, and the used visual comparison approach.

**Table 3:** Overview of all articles containing visualization techniques for comparison.

Author	Visualization technique supporting comparisons	Type of comparison	Visual comparison by Gleicher et al [23]
Kamaleswaran et al [40]^a^	Distribution: barcode plot Change over time: line Distribution: histogram	s-s^b^, s-m^c^, and s-c^d^	s-s: superposition+juxtaposition s-m: superposition+juxtaposition s-c: superposition+juxtaposition
Gomov et al [39]^a^	Change over time: calendar heat map	s-s, s-m, and s-c	s-s: juxtaposition s-m: juxtaposition s-c: juxtaposition+explicit encoding
Atherton et al [45]^a^	Change over time: Priestley timeline	s-s and s-m	s-s: juxtaposition s-m: juxtaposition
Gschwandtner et al [18]^a^	Correlation: scatterplot Change over time: columns Change over time: candlestick	s-s and s-m	s-m: juxtaposition+explicit encoding s-s: juxtaposition+explicit encoding
Tao et al [46]^a^	Change over time: Priestley timeline Flow: Sankey	s-s and s-m	s-s: juxtaposition s-m: juxtaposition
Guo et al [43]^a^	Change over time: columns Part to whole: tree maps	s-s and s-m	s-s: juxtaposition s-m: juxtaposition
Browne et al [48]	Change over time: columns Change over time: connected scatterplot	s-s	s-s: juxtaposition
Wildfire et al [34]^a^	Change over time: line	s-m	s-m: superposition
Wang et al [16]^a^	Change over time: Priestley timeline Distribution: histogram Part to whole: stacked column or bar	s-c, s-m, m-m^e^, s-s, and c-c^f^	s-c: juxtaposition s-m: juxtaposition+explicit encoding (additive)
Klimov and Shahar [15]^a^	Change over time: line	s-c and s-m	s-c: superposition s-m: superposition
Stubbs et al [35]^a^	Change over time: line	s-c and s-m	s-c: superposition s-m: superposition
Gotz and Wongsuphasawat [41]^a^	Flow: Sankey	s-c, c-c, c-m^g^	c-c: superposition
Borhani et al [38]^a^	Correlation: scatterplot	s-c	s-c: superposition
van Dortmont et al [33]^a^	—^h^	s-c	s-c: superposition+explicit encoding
Gotz and Stavropoulos [42]^a^	Correlation: bubble	c-c, s-m	c-c: superposition+explicit encoding (animation)
Dabek et al [49]^a^	Flow: network	c-c and s-c	c-c: juxtaposition s-c: juxtaposition
Dahlin et al [51]^a^	Change over time: connected scatterplot	c-c and s-c	c-c: superposition s-c: superposition
Cho et al [47]	Deviation: Surplus or deficit filled line Change over time: columns Change over time: line	c-c and m-m	c-c: juxtaposition+explicit encoding (additive)
Rogers et al [36]^a^	Change over time: line Change over Time: fan chart Change over time: Priestley timeline	c-c and s-c	c-c: juxtaposition+explicit encoding, superposition+explicit encoding s-c: juxtaposition
Nickerson et al [50]	Correlation: XY heat map	c-c	c-c: juxtaposition
Polack et al [37]	Change over time: Priestley timeline	c-c	c-c: superposition+explicit encoding (additive)
Magallanes et al [44]	Distribution: box plot Correlation: scatterplot	c-c	c-c: superposition

^a^Contain a comparison of single patients to cohorts or to multiple other patients, as visualized in Figure 2.

^b^s-s: single-to-single.

^c^s-m: single-to-multiple.

^d^s-c: single-to-cohort.

^e^m-m: multiple-to-multiple.

^f^c-c: cohort-to-cohort.

^g^c-m: cohort-to-multiple.

^h^Not available.

##### Visualizing Change Over Time: Line Charts and Timelines

This section provides a qualitative description of some of the key findings regarding the main RQ. Of the 22 articles, 5 (23%) used line charts for comparison, of which 4 (18%) specifically provided a visualization of the task of comparing single patients to multiple or a cohort of other patients. Few (2/22, 9%) of these studies used the *Priestley timelines* in addition to line charts; therefore, they applied 2 change over time techniques.

**Figure 5:**
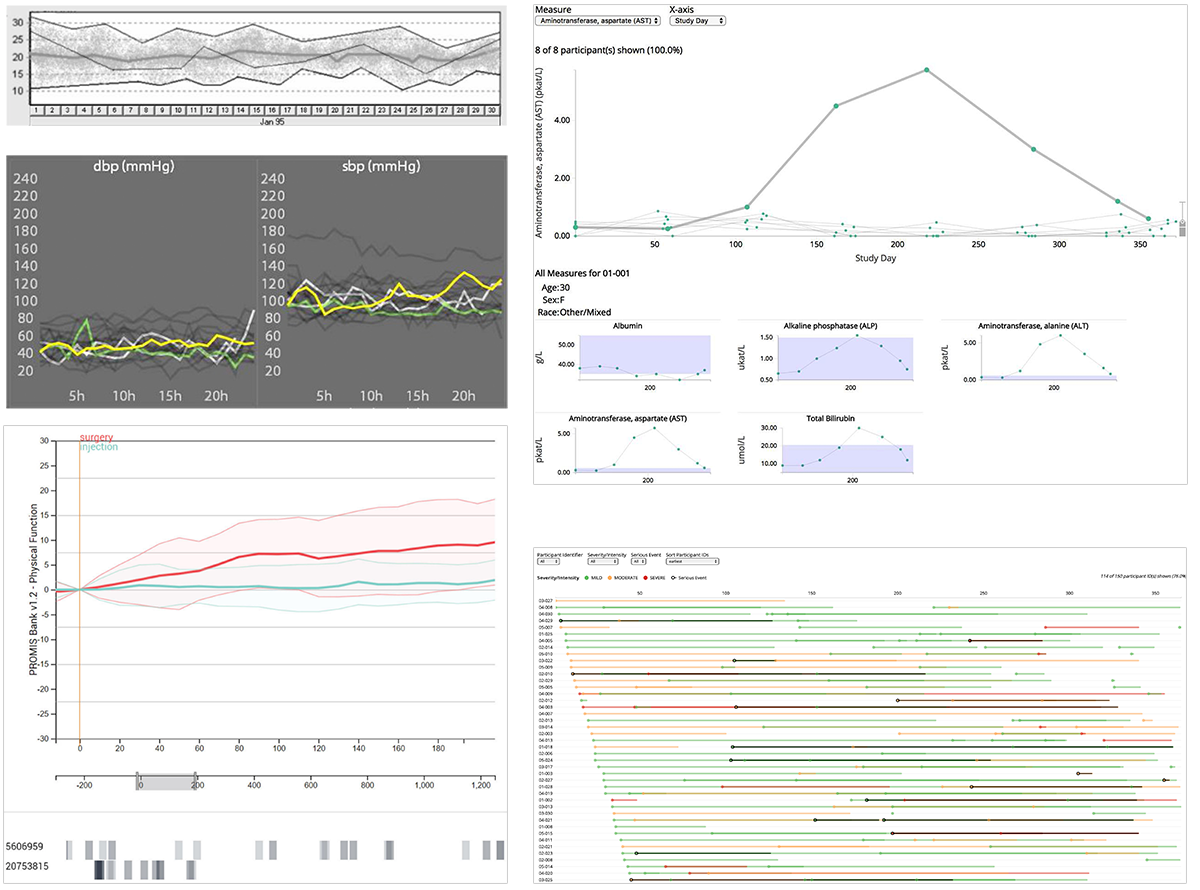
Examples showing line charts as the primary visualization technique for change over time. Use of a line chart to display the cohort (mean, maximum, and minimum boundaries) and a single selected patient (top left) (reproduced from Klimov and Shahar [15], an Open Access article). Use of a line chart to display multiple patients (unselected, selected, and queried individuals) (center left) (reproduced from Stubbs et al [35], an Open Access article). Use of a line chart to display and compare 2 cohorts (mean and quantiles) over time (bottom left). In addition, selected single patients are displayed below as Priestley timelines (reproduced from Rogers et al [36], an Open Access article, which is published under Creative Commons Attribution 4.0 International License [53]). Use of a line chart to display multiple patients (top right). In addition, small multiples (line charts) display more parameters for a selected patient (reproduced from Wildefire et al [34], with permission from Springer, conveyed through Copyright Clearance Center, Inc). Another view in the system in shows Priestley-like timelines (dot-stripe) for individual patients (bottom right) (reproduced from Wildefire et al [34], with permission from Springer, conveyed through Copyright Clearance Center, Inc).

The line chart used by Klimov and Shahar [15] demonstrated the visualization of a single concept over time; that is, the visualization of a single raw parameter (eg, carbon dioxide) over time for 1 group of patients (Figure 5, top right). The chart displays the top line for the maximal values, the bottom line for the minimal values, and the wide line (thick line) in the middle for the average values in the selected group of patients. The selected patient is displayed as an additional line, which, among other techniques, facilitates the comparison of a single patient with the cohort for this single parameter over time.

In Sim-TwentyFive by Stubbs et al [35], multiple multiline charts (arranged as small multiples) displayed various patient parameters for multiple similar patients (Figure 5, center right). Similar to the study by Klimov and Shahar [15], color coding was used to highlight the queried, selected, or most recently selected patient (green, white, or yellow, respectively), which enabled an easy comparison of the lines of interest, whereas unselected patients remained partially transparent black. In addition, aggregate polygons could be superimposed optionally to visualize the cohort mean and SD of a measure.

Similarly, Wildfire et al [34] used a multiline chart to display the development over time of multiple patients for a single selected patient measure (Figure, 5 top left). The time axis could be switched between days (starting at the baseline event of a study) or visits (the number of visits in a study). At the end of each multiline chart, a box plot representation helped compare the single selected patient line with the overall value across the cohort.

The line chart of Rogers et al [36] allowed a multitude of interactions, ranging from aggregation to normalization, serving primarily to show the development of self-reported patient outcomes over time for different cohorts and individual patients (Figure 5, bottom right). Individual patient scores could be viewed in a multiline chart, which enabled a comparison between patients. Color coding based on the calculated quartiles across the cohort could also enable individual patient comparisons with the cohort.

Composer by Rogers et al [36] and SafetyExplorer by Wildfire et al [34] used *Priestley timelines* in addition to line charts. Composer shows Patient-Reported Outcomes Measurement Information System scores over time as line charts and patient procedure code history in a *Priestley timeline*. The 2 visualizations (Figure 5, right) are time aligned with new time range selections reflected in both views. One or multiple patients can be selected in the nonaggregated line chart whose procedure code histories are shown in the *Priestly timeline*. In contrast, SafetyExplorer provides a line chart and event timeline on separate pages rather than in a coordinated manner. The suite provides all views as components.

Chronodes by Polack et al [37] explicitly showed only the use of Priestly timelines in the context of a cohort-to-cohort comparison. In detail, they used event glyphs called “kebabs” to show the occurrences of specific, differing event sequences preceding or following single or multiple shared sequences of events, called “focal events.” As the relative frequency of the preceding or following sequence was shown, this allowed the user to compare cohorts (ie, groups sharing the same focal event).

The aforementioned articles used these techniques for the task of comparing patients, and several articles also featured the same techniques but for tasks other than comparison.

For example, Borhani et al [38] also used line charts to display individual patient parameters over time but not directly for comparison. Gomov et al [39], among others, also used the Priestly timeline but only to visualize additional data, such as procedures, medication, or infections, on a per-patient basis.

##### Visualizing Distribution: Histograms

In contrast, 1 common visualization technique was used only sparingly (7/22, 32% articles) for the comparison task: the histogram. Of the 7 uses of histograms, 6 (86%) were featured in studies using single-to-cohort or single-to-multiple comparisons (Figure 2). However, only 33% (2/6) of the studies used histograms for the comparison task directly. These 2 studies, by Wang et al [16] and Kamaleswaran et al [40], used the histogram differently.

**Figure 6:**
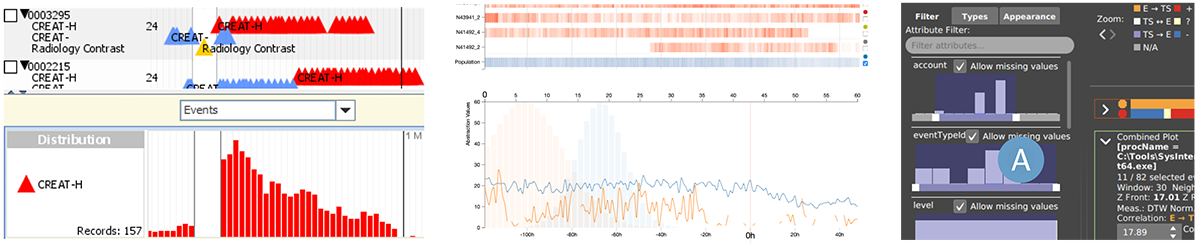
Examples of histograms to display distribution over time. Use of a histogram to display the occurrences of procedures before and after an event (left) (reproduced from Wang et al [16], with permission from IEEE). Use of histograms to display the distribution for a selected measurement of a cohort and an individual (center). Additionally, line charts display the raw data (reproduced from Kamaleswaran et al [40], with permission from the authors). Use of a histogram as an interactive filter (right) (reproduced from van Dortmont et al [33], with permission from the authors).

LifeLines2 by Wang et al [16] used an event-aligned timeline in which other types of events could be plotted as a histogram over the complete cohort (Figure 6, left). By using interactions, certain patients with events in specific regions could be selected, and a single patient’s event pattern could be directly compared with the general distribution of events, as indicated by the histogram.

Kamaleswaran et al [41] offered a detailed view of their system in which the distribution of a parameter, for example, heart rate variability, over the complete cohort was superimposed with the distribution of measured heart rate variability for a single patient (Figure 6, center). This enabled the direct comparison of a selected patient with the cohort.

The other studies that did not use histograms as a means of direct comparison used them as an auxiliary visualization, displaying additional data. For example, Gotz and Wongsuphasawat [41] used it to show the frequency of the types of interventions or medication in a selected subgroup of patients, and van Dortmont et al [33] used it as a basis for interactive filtering of the data set (Figure 6, right).

##### Visualizing Correlation: Scatterplots

The third largest group contained techniques for exploring and analyzing correlations. The 2 most frequent techniques were scatterplots with 5 occurrences and bubble charts with 2 occurrences, which extended scatterplots by additionally encoding an additional attribute to the size of the marks.

The scatterplots in the reviewed systems enabled comparison through 2 means: showing a connecting line to highlight a single patient (superpositioned) or combining them with an additional technique (juxtapositioned). Approximately 57% (4/7) of the scatterplots showed the derived data by projecting multidimensional data to 2D data [32,38], visualizing correlation as bubble size [42], or calculating a similarity value [35].

Klimov and Shahar [15] visualized the measurement of a parameter over time in a group of patients. Here, the diagram showed the measurements for multiple patients without visual distinction for different patients. When a patient was selected, all measurements were connected by a line. In this way, a single patient could be visually compared with a group of patients.

**Figure 7:**
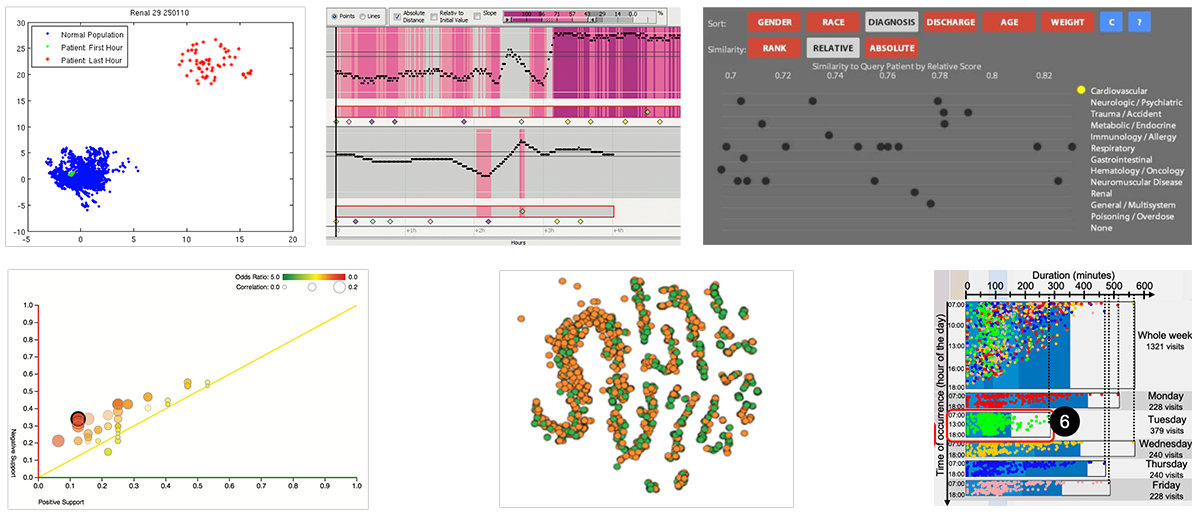
Examples of scatterplots to display correlation. Use of a scatterplot to display a 2D projection of values of a cohort and a single patient (top left) (reproduced from Borhani et al [38], with permission from IEEE). Use of a scatterplot to display measurements over time for multiple patients (top center) (reproduced from Gschwandtner et al [18], with permission from IEEE). A scatterplot to show similarity scores across multiple patients (top right) (reproduced from Stubbs et al [35], an Open Access article). Use of a scatterplot (bubble chart) to display the positive and negative outcome contributions of a selected sequence of procedures (bottom left) (reproduced from Gotz et al [42], with permission from the authors). A scatterplot to display a 2D projection of event co-occurrences (bottom center) (reproduced from Guo et al [43], with permission from IEEE). A scatterplot to display occurrences and duration of consultation events (bottom right) (reproduced from Magallanes et al [44], with permission from IEEE).

Borhani et al [38] projected a 4D model onto a 2D plane (Figure 7, top left). The measurements of multiple patients in the “normal” state were shown as a cluster of blue dots. The measurements of the first and last hours of a selected patient were shown within the scatterplot in green and red, respectively. This allowed for quick identification of normal and abnormal measurements of the patient. In addition, the original (ie, nonprojected) measurements of the selected patients were shown in line charts juxtapositioned below.

CareCruiser (Gschwandter et al [18]) showed the parameters over time for multiple patients under investigation (Figure 7, top center). For each patient, a chart visualized the parameter’s values over time to view their condition. The time axes were relative to a specified time point; thus, the vertically juxtapositioned charts enabled a direct comparison. Different color-coded bands eased visually identifying relevant events of the patient’s development.

The Sim-TwentyFive visualization system [35] enabled querying and comparing episodes and measurements of a selected patient with the 25 most similar other patients (Figure 7, top right). A “cartesian coordinate plot” mapped a calculated score to the x-axis such that the distance to the selected patient indicates their similarity for different measures. Users could switch between different continuous and categorical parameters along the y-axis. The similarity index allowed viewers to compare selected patients with multiple others.

DecisionFlow [42] aggregated event sequences into milestones and intermediate episodes, resulting in visually less complex sequences. DecisionFlow contained a statistical panel with a bubble chart as the main visualization (Figure 7, bottom left). The bubble chart enabled the comparison of events over time and the identification of relevant events for further exploration. Each circle represents an event type, positioned onto 2 axes representing positive or negative support; that is, “the fraction of intermediate episodes in the positive [resp. negative] outcome group containing one or more occurrences of the event type.” Its size encoded the correlation, with the additional color showing an odds ratio consistent with all other color codings within the visualization system. The correlation and odds ratios were based on the positive and negative outcome groups. Thus, circles closer to the x-axis represented event types that appeared more often in episodes with a positive outcome and vice versa.

Guo et al [43] presented color-coded circles on a 2D chart to support a visual comparison of event co-occurrence (Figure 7, bottom center). It visualized multiple dimensions on a 2D plane using a dimension reduction technique. This dimension reduction approach resulted in similar events being closer together and dissimilar events being more distant in this chart. Although the used t-distributed stochastic neighbor embedding projection often seemed to show clusters, it is heavily dependent on the chosen parameters of the algorithm. The position of each individual event on the x- and y-axes is semantically ambivalent, and thus, this view is only tangentially related to classic scatterplots.

The scatterplot in the study by Magallanes et al [44] enabled the comparison of different weekdays, event sequences, and event occurrences (Figure 7, bottom right). Although it did not facilitate single-to-cohort or single-to-multiple comparisons, it was an unusual approach for visualizing a parameter over time (ie, the occurrence and duration of consultation events). The scatterplot was shown as a superposition of the scatterplots for different patients. This allowed for the quick identification of normal and abnormal measurements. Although the data points can be identified as outliers, the user cannot identify the patient the event occurrence belongs to.

The presented examples of individual results demonstrate common approaches for comparing time-oriented data. Most applied techniques are line charts that show the development of a parameter over time for single or multiple individuals or aggregated for cohorts. Priestley timelines in the presented cases show the periods and mark the start and end of an episode type to be compared but not for directly comparing the quantities.

Bar charts and histograms display the distribution over time and are often used as interactive charts for filtering.

Scatterplots have diverse applications, from simple dots over time to more complex techniques that show correlations between patients and parameters.

## Discussion

### Summary of Evidence

#### Methodology

The first screening step was conducted by a diverse interdisciplinary team, with contributors having different levels of expertise in visualization research. The last screening and analysis steps were performed by 4 experts from the core team. Although experience and expertise in visualization were advanced, many issues arose during the extraction of data items when applying the different taxonomies. We are aware that in some of our extraction steps, the interpretation of the presented visualizations and application of the corresponding taxonomies may vary. Although we discussed debatable data items, other individuals may obtain different results in some cases.

To provide a systematic overview of the visualization techniques, we investigated different existing taxonomies and classification schemas. We chose Visual Vocabulary as it structures the techniques according to the main objectives. In addition to the task classification by Munzner [6], we collected techniques and their visual analysis objectives and tasks. We found it beneficial to have experts and incorporate publications from both the medical and visualization fields. Through the combination of taxonomies from practice and academia, we were able to collect and review the types of visualizations used for the specific task of comparing temporal patient data. In this way, we could provide an overview of the different visualization techniques and the contexts in which they are used (RQ2).

On a secondary note, we find it worthwhile to highlight how the 2 communities may learn from each other. State-of-the-art reports (STARs) are a major approach to systematically reviewing specific fields in information visualization (McNabb and Laramee [54] and Wang and Laramee [2]). Although they are similarly rigorous in their approach, there is no standardized methodology for collecting and documenting evidence in information visualization reviews. In contrast, STAR articles often use visualizations to summarize their findings. Thus, there might be 2 promising targets for information visualization researchers to build more standardized reviewing and survey procedures and for medical informatics researchers to embrace some of the visual summaries that STAR articles use.

To provide readers with an interactive way of exploring the visualization systems from our scoping review, we created a visual literature browser using the SurVis software [55]. Our tool not only provides a selection of attributes to see the use of specific visualization techniques but also enables cross-filtering to identify systems combining a set of attributes such as medical context, visualization, and patient entities. Our companion tool is available on the web [56].

#### Medical Characteristics

As synthesized previously, most of the reviewed studies were in the field of clinical research. We assume this to be because of higher data quality and availability in clinical research, in contrast to data from clinical care, where data are often stored in legacy systems and are not necessarily standardized. A recent survey on EHR visualizations confirmed this assumption; the authors identified 3 challenges impeding the use of EHR data: accessibility, data quality, and interoperability [2].

With respect to the abstracted MeSH terms for coding the diseases, and leaving the generic category of “Pathological Conditions, Signs and Symptoms” aside, we observed a rather wide spread of diseases targeted in the visualization systems. From a medical perspective, this seems to be unexpected, as tumors and cardiovascular diseases are more common. However, from an opportunistic perspective, in selected medical areas, more data are often digitized and easily available, which might result in higher use frequency in medical informatics studies.

This increasing availability of data from primary care facilities enables secondary use in the field of clinical research. In the reviewed studies, we identified the treatment outcome as the major objective of analysis, stemming from both clinical research and clinical care (RQ2). Overall, this emphasizes the need to visually compare changes over time, distributions, and correlations between individuals and their cohorts.

#### Visualizations

The visual analysis objective most supported in the reviewed systems was to show changes over time. This observation matched our expectations, as the review focused on temporal patient data. Although many medical data have a temporal component, not all visualizations in the medical field focus on time. A scoping review on public health visualizations [12] identified visually analyzing spatial patterns as the most common objective (43.6%), with change over time coming in a distant second, at 14.5%.

To visually investigate correlations, scatterplots and bubble charts were identified as the most common. Here, we noticed that some systems use scatterplots in a nontraditional manner, as they plot a parameter over time on 1 axis [16,19,43]. Although time is a continuous scale and, thus, fits the definition of scatterplots, a more common technique for showing a continuous measure over time is a line chart. When the dots are not sequentially ordered on 1 of the 2 axes but by the values of a nontemporal measure, a connected scatterplot could be used. Both the line chart and connected scatterplot were from the change over time category. We can only assume that the choice of scatterplots (or, perhaps more precisely, scattered dots over time) was because of the design goal of having less cluttered views by omitting the lines. This exemplifies how visualization techniques typically not used for temporal data are used in such ways.

Owing to the nature of single or individual patient data, simple visualization techniques are being used, and the same applies to multiple patients as well, up to a certain degree. In the case of cohorts, which are most often represented as 1D data (aggregated on value and or on time), the same applies and the basic techniques are the most used.

The reported visualization techniques are part of the visualization systems or prototypes of varying maturity levels. Some more advanced and highly interactive systems with a variety of views combined a multitude of techniques, whereas others presented only 1 single and static visualization for 1 objective. We did not evaluate this characteristic and therefore considered the maturity level (complexity of the system, variety of use cases, and tasks) as an interesting parameter for future work. Some articles were simple mock-ups (eg, showing a prototype of a user interface). Other presented articles were edge cases in the sense that the application of the visualization system was primarily developed outside the health care domain, and its application to patient data was shown as a potential use case (eg, ChronoCorrelator showing a use case for analyzing event threads on a server).

#### Comparison

We identified single patient, multiple patients, and cohort as the entities to visually compare and collected visualization techniques supporting the comparison of any of their combinations. As the reduction of the original search results allowing the comparison of different single patients to the results and the comparison of a single patient to a cohort or multiples was quite noticeable (from 57 to 22), we retained a subset of the studies that would have been dropped at this stage. Thus, these studies (Figure 2) were analyzed in the same manner as the studies explicitly allowing the targeted task. Although these studies might not have been specifically designed to allow the comparison of a single patient with multiple patients or cohorts, the used techniques themselves seemed to be capable of such tasks with little modification. This shows that (1) the visual comparison of a single patient with multiple other patients (single-to-multiple and single-to-cohort) is relatively underdeveloped in comparison with single-to-single or cohort-to-cohort and (2) many existing visualizations purpose-built for the comparison of cohorts among themselves or of a single patient with another individual patient could be adapted to further combinations as well.

**Figure 8:**
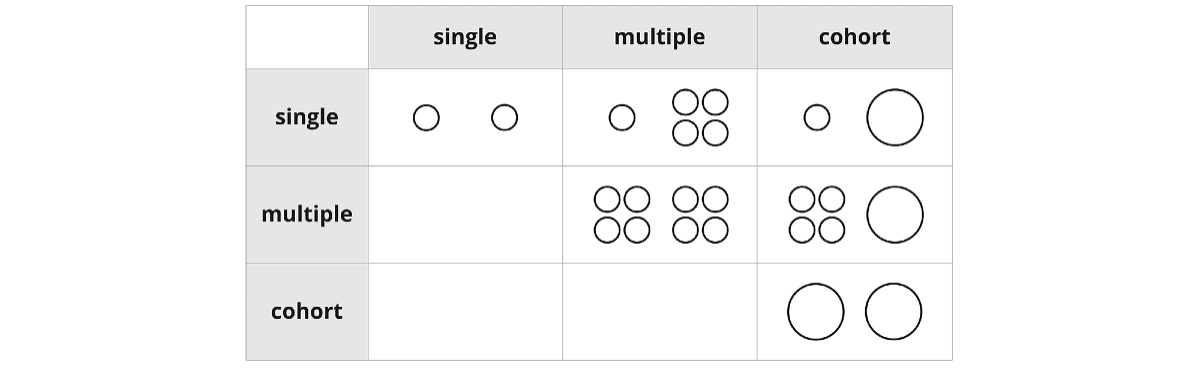
Schematic overview of the possible comparisons between different patient entities ranging from single patient to multiple patients and cohorts. Multiple-to-cohort emphasizes the distinction between the visual representation of multiple patients versus an aggregated view of the cohort.

By applying our taxonomy for detailed identification of the comparison aspect, we introduced the differentiation between single-to-single, single-to-multiple, single-to-cohort, cohort-to-cohort, multiple-to-multiple, and multiple-to-cohort. Although this differentiation may seem trivial with respect to set theory, it reveals the not directly obvious disruption between showing multiple individuals or a group to be considered the opposing entity of the comparison (Figure 8).

When visualizing patients, we identified the difference between multiple patients and a cohort not in the size of the group, but in the fact that visualizing cohorts requires aggregation of the data beforehand. As shown in our review, this typically goes hand in hand with a different visual representation. For showing a measurement over time, a way of representing the cohort is by visualizing the central tendency (eg, mean) and spread (eg, range) as different lines. An alternative would be to select fitting temporal windows and visualize the spreads of the measurement per time range as box plots.

**Figure 9:**
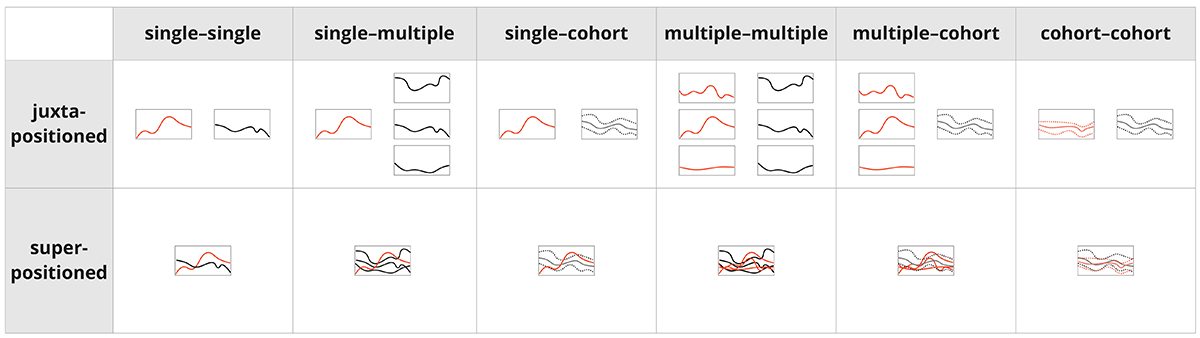
All possible combinations for comparing 1 measurement over time between different patient entities (single patient, multiple patients, and cohorts) in the case of line charts. For single and multiple patients, each line represents 1 measurement, whereas, for cohorts, the chart represents the mean and range. All combinations are shown in juxtapositioned and superpositioned layouts, with the colors supporting legibility in the latter.

In Figure 9, we show all possible combinations of visually comparing measurements over time between different patient entities. Figure 9 exemplifies this for line charts, whereas the conceptual space of the different comparison combinations is agnostic to the used visualization technique. In addition, we only show variations for juxtapositioned versus superpositioned layouts, whereas a wide range of alternate options such as interactions exist. Choosing the appropriate visualization, layout position, and interaction is a major challenge in designing visual analysis systems and requires human-centric development approaches to match the visualizations with the tasks and requirements of users. Overall, although this visual example using line charts provides some initial hints into what might work better than other combinations (eg, superpositioned multiple-to-multiple comparisons seem to be visually overly complex), it is an early exploration of a design space.

### Limitations

Although we did not restrict our search to journals and included conference proceedings as these are one of the primary types of publications in the computer science field, we found only a small number of articles that matched all criteria. As the importance of visual analytics in general and visual analytic systems in particular continues to grow, we expected to include more articles from recent years; however, only a few were identified to match our criteria. As described earlier, we iteratively refined our search terms in collaboration with an experienced librarian, and therefore, we assume this to be because of the particular combination of patient-to-cohort comparisons and visualizations focusing on time-oriented data. However, we are aware that we may have missed relevant works; for example, systems that could be primarily cohort visualization tools might also support some detailed highlighting of individual patients without mentioning this explicitly or discussing it in their written report.

We extracted and synthesized a wide set of relevant attributes to summarize the major characteristics of the reviewed studies. However, there is a range of further investigations that we found to be outside the scope of this review. Although we took various specifics of the data into account, we did not evaluate data preparation or data transformation steps alone if they were not an essential aspect of the used visualization technique (such as showing high-dimensional data in a 2D display).

Studies on visual analysis systems usually gather feedback through usability evaluations or demonstrate its applicability through case studies. Although we did not synthesize such attributes in our scoping review, we acknowledge the importance of understanding user feedback to properly assess the usefulness of visualization systems and emphasize the need for further research in this regard.

In addition to the authors and publication year, we restricted the metadata extraction to information about the publication outlet. Analyzing this would allow us to explore the correlations between the extracted attributes and the research area. For instance, one could investigate whether visualization researchers use more complex visualization techniques than researchers in the medical field. This could not be covered in this review, and we did not incorporate it into the analysis.

Furthermore, we did not fully synthesize combinations of the extracted attributes. For instance, it might be insightful to further examine the kinds of interactions that are offered more frequently for specific visualization techniques. The investigation of this explicit combination could lead to a better understanding of which selection techniques for 1 or multiple patients with specific characteristics are appropriate for different aggregated cohort visualizations. Although the articles were analyzed to include visualization of task-specific actions and targets, in this review, it could not be evaluated in further detail whether specific action-target pairs appeared more or less frequently. However, the analysis of these pairs could lead to interesting RQs in the field of visualization research. Our web-based companion tool at [55] provides the first basic possibility of exploring combinations of extracted attributes such as medical diseases and visualization techniques.

### Conclusions

Visual analytic systems mitigate the complexity of time-oriented patient data through data analysis and interactive visualizations by facilitating attention to underlying and hidden patterns. In this scoping review, we examined the available literature and identified and clustered visualization techniques that specifically supported the task of comparing time-oriented patient data (RQ1). We collected and reported on the visual analysis objectives and tasks with a specific focus on the range of options to compare individual patients with multiple patients or with a cohort (RQ2). Finally, we surveyed and presented the medical characteristics, data type categories, and interaction techniques of the reviewed visualization systems (RQ3).

As this work is a scoping review, we consider the identified articles and the performed extraction steps as the first step for conducting further research in the form of a more advanced extraction. We found that a small set of publications specifically contained single-to-multiple or single-to-cohort comparison and provided visualizations to support this task. In most cases, we also found that basic visualization techniques such as line charts, event timelines, histograms, or scatterplots were used efficiently. Time-oriented comparisons between a single patient and multiple patients or a cohort are mostly used for laboratory and vital sign parameters, followed by analysis and comparison of procedures and diagnoses. We identified many potentially interesting approaches and deemed many of these techniques to be applicable for a comparison of single patients with multiple patients and cohorts through small adaptations.

We anticipate that we have convincingly argued for the usefulness of visually comparing individual patients with cohorts and encourage researchers to further investigate visualization and interaction techniques for such comparisons. Finally, our review showed the need to systematically review further systems and techniques to propose a proper design space for comparing the temporal data of single, multiple, and cohort patients.

## Appendix

Multimedia Appendix 1 Search queries on literature databases and the number of results.

Multimedia Appendix 2 Extraction table with data items for the sources of evidence.

## Abbreviations

EHR: electronic health record
KNAVE: Knowledge-based Navigation of Abstractions for Visualization and Explanation
MeSH: Medical Subject Headings
PRISMA: Preferred Reporting Items for Systematic Reviews and Meta-Analyses
PRISMA-ScR: Preferred Reporting Items for Systematic Reviews and Meta-Analyses extension for Scoping Reviews
PROSPERO: International Prospective Register of Systematic Reviews
RQ: research question
STAR: state-of-the-art report

## References

[1] Shneiderman B, Plaisant C, Hesse BW (2013). Improving healthcare with interactive visualization. Computer.

[2] Wang Q, Laramee RS (2022). EHR STAR: the state‐of‐the‐art in interactive EHR visualization. Comput Graphics Forum.

[3] Zhang Z, Gotz D, Perer A (2014). Iterative cohort analysis and exploration. Inf Vis.

[4] Aigner W, Miksch S, Schumann H, Tominski C (2011). Visualization of Time-Oriented Data.

[5] Brehmer M, Munzner T (2013). A multi-level typology of abstract visualization tasks. IEEE Trans Visual Comput Graphics.

[6] Munzner T (2014). Visualization Analysis and Design.

[7] Miksch S, Aigner W (2014). A matter of time: applying a data–users–tasks design triangle to visual analytics of time-oriented data. Comput Graph.

[8] Preim B, Lawonn K (2020). A survey of visual analytics for public health. Comput Graphics Forum.

[9] Rind A, Wang TD, Aigner W, Miksch S, Wongsuphasawat K, Plaisant C, Shneiderman B (2011). Interactive Information Visualization to Explore and Query Electronic Health Records.

[10] Thomas JJ, Cook KA (2005). Illuminating the Path: The Research and Development Agenda for Visual Analytics.

[11] Keim DA, Mansmann F, Schneidewind J, Thomas J, Ziegler H, Simoff SJ, Böhlen MH, Mazeika A (2008). Visual analytics: scope and challenges. Visual Data Mining.

[12] Plaisant C, Milash B, Rose A, Widoff S, Shneiderman B (1996). LifeLines: visualizing personal histories. Proceedings of the SIGCHI Conference on Human Factors in Computing Systems.

[13] Shahar Y, Cheng C (1998). Knowledge-based visualization and navigation of time-oriented clinical data and their abstractions. Proceedings of the 13th European Conference on Artificial Intelligence - Intelligent Data Analysis in Medicine and Pharmacology (IDAMAP) Workshop.

[14] Shahar Y, Goren-Bar D, Boaz D, Tahan G (2006). Distributed, intelligent, interactive visualization and exploration of time-oriented clinical data and their abstractions. Artif Intell Med.

[15] Klimov D, Shahar Y (2005). A framework for intelligent visualization of multiple time-oriented medical records. AMIA Annu Symp Proc.

[16] Wang TD, Plaisant C, Shneiderman B, Spring N, Roseman D, Marchand G, Mukherjee V, Smith M (2009). Temporal summaries: supporting temporal categorical searching, aggregation and comparison. IEEE Trans Vis Comput Graph.

[17] Monroe M, Lan R, Lee H, Plaisant C, Shneiderman B (2013). Temporal event sequence simplification. IEEE Trans Vis Comput Graph.

[18] Gschwandtner T, Aigner W, Kaiser K, Miksch S, Seyfang A (2011). CareCruiser: exploring and visualizing plans, events, and effects interactively. 2011 IEEE Pacific Visualization Symposium.

[19] Birch N, Schulz C, Peter J, Klingert W, Schenk M, Weiskopf D, Krone M (2020). Visual analysis of multivariate intensive care surveillance data. Eurographics Workshop on Visual Computing for Biology and Medicine.

[20] Guo Y, Guo S, Jin Z, Kaul S, Gotz D, Cao N (2021). A survey on visual analysis of event sequence data. IEEE Trans Vis Comput Graph (forthcoming).

[21] West VL, Borland D, Hammond WE (2015). Innovative information visualization of electronic health record data: a systematic review. J Am Med Inform Assoc.

[22] Rostamzadeh N, Abdullah SS, Sedig K (2021). Visual analytics for electronic health records: a review. Informatics.

[23] Gleicher M, Albers D, Walker R, Jusufi I, Hansen CD, Roberts JC (2011). Visual comparison for information visualization. Inf Vis.

[24] Wicks P, Massagli M, Frost J, Brownstein C, Okun S, Vaughan T, Bradley R, Heywood J (2010). Sharing health data for better outcomes on PatientsLikeMe. J Med Internet Res.

[25] Tricco AC, Lillie E, Zarin W, O'Brien KK, Colquhoun H, Levac D, Moher D, Peters MD, Horsley T, Weeks L, Hempel S, Akl EA, Chang C, McGowan J, Stewart L, Hartling L, Aldcroft A, Wilson MG, Garritty C, Lewin S, Godfrey CM, Macdonald MT, Langlois EV, Soares-Weiser K, Moriarty J, Clifford T, Tunçalp Ö, Straus SE (2018). PRISMA extension for scoping reviews (PRISMA-ScR): checklist and explanation. Ann Intern Med.

[26] (2021). chart-doctor/visual-vocabulary at main. GitHub.

[27] Tennekes M, Chen M (2021). Design space of origin‐destination data visualization. Comput Graphics Forum.

[28] Borkin MA, Vo AA, Bylinskii Z, Isola P, Sunkavalli S, Oliva A, Pfister H (2013). What makes a visualization memorable?. IEEE Trans Vis Comput Graph.

[29] Wilke CO (2019). Fundamentals of Data Visualization: A Primer on Making Informative and Compelling Figures.

[30] Theis S, Rasche PW, Bröhl C, Wille M, Mertens A (2018). Task-data taxonomy for health data visualizations: web-based survey with experts and older adults. JMIR Med Inform.

[31] Rostamzadeh N, Abdullah SS, Sedig K (2020). Data-driven activities involving electronic health records: an activity and task analysis framework for interactive visualization tools. Multimodal Technol Interact.

[32] Guo S, Xu K, Zhao R, Gotz D, Zha H, Cao N (2018). EventThread: visual summarization and stage analysis of event sequence data. IEEE Trans Vis Comput Graph.

[33] van Dortmont MA, van den Elzen S, van Wijk JJ (2019). ChronoCorrelator: enriching events with time series. Comput Graphics Forum.

[34] Wildfire J, Bailey R, Krouse RZ, Childress S, Sikora B, Bryant N, Rosanbalm S, Wilson E, Modell JG (2018). The safety explorer suite: interactive safety monitoring for clinical trials. Ther Innov Regul Sci.

[35] Stubbs B, Kale DC, Das A (2012). Sim•TwentyFive: an interactive visualization system for data-driven decision support. AMIA Annu Symp Proc.

[36] Rogers J, Spina N, Neese A, Hess R, Brodke D, Lex A (2019). Composer-visual cohort analysis of patient outcomes. Appl Clin Inform.

[37] Polack Jr PJ, Chen ST, Kahng M, DE Barbaro K, Basole R, Sharmin M, Chau DH (2018). Chronodes: interactive multifocus exploration of event sequences. ACM Trans Interact Intell Syst.

[38] Borhani Y, Fleming S, Clifton DA, Sutherland S, Hills L, Meredith D, Pugh CW, Tarassenko L (2010). Towards a data fusion model for predicting deterioration in dialysis patients. Proceedings of the 2010 Computing in Cardiology.

[39] Gomov M, Chou JK, Li JK, Sen S, Cho K, Tran N, Ma KL (2017). Aiding infection analysis and diagnosis through temporally-contextualized matrix representations. Proceedings of the 2017 IEEE Workshop on Visual Analytics in Healthcare.

[40] Kamaleswaran R, James A, Collins C, McGregor C (2016). CoRAD: visual analytics for cohort analysis. Proceedings of the 2016 IEEE International Conference on Healthcare Informatics.

[41] Gotz D, Wongsuphasawat K (2012). Interactive intervention analysis. AMIA Annu Symp Proc.

[42] Gotz D, Stavropoulos H (2014). DecisionFlow: visual analytics for high-dimensional temporal event sequence data. IEEE Trans Vis Comput Graph.

[43] Guo S, Jin Z, Gotz D, Du F, Zha H, Cao N (2019). Visual progression analysis of event sequence data. IEEE Trans Vis Comput Graph.

[44] Magallanes J, van Gemeren L, Wood S, Villa-Uriol MC (2019). Analyzing time attributes in temporal event sequences. Proceedings of the 2019 IEEE Visualization Conference.

[45] Atherton PJ, Jasperson B, Nibbe A, Clement-Brown KA, Allmer C, Novotny P, Erlichman C, Sloan JA (2003). What happened to all the patients? Event charts for summarizing individual patient data and displaying clinically significant changes in quality of life data. Drug Information J.

[46] Tao C, Wongsuphasawat K, Clark K, Plaisant C, Shneiderman B, Chute CG (2012). Towards event sequence representation, reasoning and visualization for EHR data. Proceedings of the 2nd ACM SIGHIT International Health Informatics Symposium.

[47] Cho M, Kim B, Bae HJ, Seo J (2014). Stroscope: multi-scale visualization of irregularly measured time-series data. IEEE Trans Vis Comput Graph.

[48] Browne SH, Behzadi Y, Littlewort G (2015). Let visuals tell the story: medication adherence in patients with type II diabetes captured by a novel ingestion sensor platform. JMIR Mhealth Uhealth.

[49] Dabek F, Chen J, Garbarino A, Caban JJ (2015). Visualization of longitudinal clinical trajectories using a graph-based approach. Proceedings of the 2015 Workshop on Visual Analytics in Healthcare.

[50] Nickerson PV, Baharloo R, Wanigatunga AA, Manini TM, Tighe PJ, Rashidi P (2018). Transition icons for time-series visualization and exploratory analysis. IEEE J Biomed Health Inform.

[51] Dahlin S (2020). Exploring the usefulness of Lexis diagrams for quality improvement. BMC Med Inform Decis Mak.

[52] Home of exploratory data analysis. Keshif.

[53] Attribution 4.0 International (CC BY 4.0). Creative Commons.

[54] McNabb L, Laramee RS (2017). Survey of Surveys (SoS) ‐ mapping the landscape of survey papers in information visualization. Computer Graphics Forum.

[55] Beck F, Koch S, Weiskopf D (2016). Visual analysis and dissemination of scientific literature collections with SurVis. IEEE Trans Vis Comput Graph.

[56] Visualization techniques of time-oriented data for the comparison of single patients to multiple patients or cohorts. GitHub.

