# Supplementary material for: Visualization Techniques of Time-Oriented Data for the Comparison of Single Patients With Multiple Patients or Cohorts: Scoping Review
Source: J Med Internet Res. 2022 Oct 24;24(10):e38041. doi: 10.2196/38041 (PMC9641521; doi:10.2196/38041)
Supplement: Multimedia Appendix 1 [file jmir_v24i10e38041_app1.docx]

**Multimedia Appendix 1**

Search strings and results (directly on literature-database):

1. PubMed: # 396

   ( "temporal data"[tiab] OR "temporal sequence*"[tiab] OR "temporal pattern*"[tiab] OR "temporal abstraction*"[tiab] OR "temporal event*"[tiab] OR "time sequence*"[tiab] OR "time series"[tiab] OR "time period*"[tiab] OR "time frame*"[tiab] OR "timeframe*"[tiab] OR timeline*[tiab] OR time-oriented[tiab] OR ("time"[tiab] AND "events"[tiab]) ) AND ( visuali*[tiab] OR "visual analy*"[tiab] ) AND ( data[tiab] OR information[tiab] ) AND ( patient[tiab] OR patients[tiab] OR "health care"[tiab] OR healthcare[tiab] OR cohort*[tiab] OR "electronic health record*"[tiab] )
2. IEEE Explore: 

   # 243

   ( "temporal data" OR "temporal sequence" OR "temporal pattern" OR "temporal abstraction" OR "temporal event*" OR "time sequence" OR "time series" OR "time period" OR "time frame" OR "timeframe" OR Timeline OR Time-oriented OR ("time" AND "events") ) AND ( visuali* OR “visual analy*“ ) AND ( Data OR Information ) AND ( Patient OR patients OR "health care" OR healthcare OR cohort* OR "electronic health record*" )


   *#241*

   *( "temporal data" OR "temporal sequences" OR "temporal patterns" OR "temporal abstractions" OR "temporal event*" OR "time sequences" OR "time series" OR "time periods" OR "time frames" OR "timeframes" OR Timelines OR Time-oriented OR ("time" AND "events") ) AND ( visuali* OR “visual analy*“ ) AND ( Data OR Information ) AND ( Patient OR patients OR "health care" OR healthcare OR cohort* OR "electronic health record*" )*
3. Web Of Science:

   #463 (Web of Science Core Collection) & in English

   TS=( ( "temporal data"   OR  "temporal sequence*"   OR "temporal pattern*"   OR  "temporal abstraction*"   OR  "temporal event*"  OR  "time sequence*"   OR  "Time series"   OR  "time period*"   OR  "time frame*"   OR  "timeframe*"   OR "Timeline*"   OR  "Time-oriented"   OR (**"time" AND event***) )   AND  ( visuali*  OR “visual analy*“ )   AND  ( Data  OR Information )   AND  ( Patient  OR patients   OR "health care"  OR healthcare   OR cohort*  OR "electronic health record*" ) )
4. ACM DL: 

   # 52 
   ( Abstract:(“temporal data”) OR Title:(“temporal data”) OR Abstract:(“temporal sequence”) OR Title:(“temporal sequence”) OR Abstract:(“temporal sequences”) OR Title:(“temporal sequences”) OR Abstract:(“temporal pattern”) OR Title:(“temporal pattern”) OR Abstract:(“temporal patterns”) OR Title:(“temporal patterns”) OR Abstract:(“temporal abstraction”) OR Title:(“temporal abstraction”) OR Abstract:(“temporal abstractions”) OR Title:(“temporal abstractions”) OR Abstract:(“temporal event”) OR Title:(“temporal event”) OR Abstract:(“temporal events”) OR Title:(“temporal events”) OR Abstract:(“time sequence”) OR Title:(“time sequence”) OR Abstract:(“time sequences”) OR Title:(“time sequences”) OR Abstract:(“time series”) OR Title:(“time series”) OR Abstract:(“time period”) OR Title:(“time period”) OR Abstract:(“time periods”) OR Title:(“time periods”) OR Abstract:(“time frame”) OR Title:(“time frame”) OR Abstract:(“time frames”) OR Title:(“time frames”) OR Abstract:(timeframe*) OR Title:(timeframe*) OR Abstract:(timeline*) OR Title:(timeline*) OR Abstract:(“time-oriented”) OR Title:(“time-oriented”) OR ((Abstract:(“time”) AND Abstract:(event*)) OR (Title:(“time”) AND Title:(event*))) ) AND ( Abstract:(visuali*) OR Title:(visuali*) OR Abstract:(“visual analysis”) OR Title:(“visual analysis”) OR Abstract:(“visual analyzing”) OR Title:(“visual analyzing”) OR Abstract:(“visual analysing”) OR Title:(“visual analysing”) OR Abstract:(“visual analytics”) OR Title:(“visual analytics”) ) AND ( Abstract:(data) OR Title:(data) OR Abstract:(information) OR Title:(information) ) AND ( Abstract:(patient) OR Title:(patient) OR Abstract:(patients) OR Title:(patients) OR Abstract:(“health care”) OR Title:(“health care”) OR Abstract:(healthcare) OR Title:(healthcare) OR Abstract:(cohort*) OR Title:(cohort*) OR Abstract:(“electronic health record”) OR Title:(“electronic health record”) OR Abstract:(“electronic health records”) OR Title:(“electronic health records”) )
